# Conducting a conventional multi-casualty incident in COVID-19 personal protective equipment - a semi-structured interview

**DOI:** 10.1186/s13049-021-00838-w

**Published:** 2021-01-27

**Authors:** James Raitt, Ben Watts, Jaspreet Rayet, Mark Hodkinson, David Zideman

**Affiliations:** Thames Valley Air Ambulance, Stokenchurch House, Oxford Rd, Stokenchurch, High Wycombe, HP14 3SX UK

**Keywords:** COVID-19, Multi casualty incident, Personal Protective Equipment

## Abstract

**Background:**

The risk of COVID-19 transmission to healthcare professionals is likely to continue for the foreseeable future. The wearing of personal protective equipment (PPE) presents a number of potential challenges to responders that may impact upon the management of patients in a multi-casualty incident.

This report describes a multi-agency multi-casualty incident. It identifies learning points specifically related to the challenges of conducting a conventional multi-casualty incident in COVID-19 PPE.

**Case:**

The multi casualty incident in Reading, UK on the 20 June 2020 involved six stab injury victims and was attended by four pre-hospital critical care teams. This was the first conventional multi-casualty incident that pre-hospital critical care teams had attended during the COVID-19 era and it was conducted in COVID-19 PPE (1). The scene was an urban park where three patients were confirmed to be in Traumatic Cardiac Arrest (TCA) from stab wounds and another three patients had also suffered stab injuries. By the time the incident had concluded three patients were pronounced dead at the scene. Two patients were transported to the local trauma unit and one patient was transported to the regional Major Trauma Centre depending on the severity of their injuries.

**Conclusions:**

We conducted a semi structured telephone interview with the critical care clinicians who were involved in the incident. The interviews focused specifically on the challenges of responding whilst wearing COVID-19 PPE, rather than the wider challenges of responding to such an incident. The key learning points identified were:

- Improving the identifiability of clinicians in level 3 PPE
  - wearing identification tabards
  - using visible labelling on PPE suits
- Improving communication by radio
  - using a belt to carry the radio
  - using an earpiece and push to talk system.
- Training in conducting multi casualty incidents in level 3 PPE.

## Background

The multi-casualty incident in Reading on the 20 June 2020 was the first conventional incident that multiple pre-hospital critical care teams had attended during the COVID-19 era. It was conducted in COVID-19 PPE [1]. The incident involved six stab injury victims and was attended by South Central Ambulance Service NHS Trust (SCAS) and pre-hospital critical care teams from Thames Valley Air Ambulance (TVAA), Hampshire and Isle of Wight Air Ambulance (HIOWAA) and London’s Air Ambulance (LAA) as well as the British Association for Immediate Care (BASICS).

The risk of COVID-19 transmission to healthcare professionals is likely to continue for the foreseeable future and the wearing of PPE presents a number of potential challenges to responders that may impact upon the management of casualties at multi-casualty incidents.

This report identifies learning points specifically related to the challenges of conducting clinical care at a conventional multi-casualty incident in COVID-19 PPE. We hope that this study will assist other services to prepare and train for, and respond to multi casualty incidents whilst wearing COVID PPE.

## Case

At 18:57 on 20 June 2020 the first of approximately ten emergency calls were received at SCAS Emergency Operations Centre (EOC). The TVAA critical care paramedic (CCP) working on the Helicopter Emergency Medical Services (HEMS) Dispatch Desk, based within the EOC, listened passively to the call and made the decision to dispatch multiple critical care resources. The call suggested that there were several casualties in traumatic cardiac arrest (TCA) and up to ten casualties who had sustained penetrating stab injuries. Two TVAA pre-hospital critical care teams were dispatched. The first was a dual CCP asset in a fast response car and the second, comprising a pre-hospital emergency medical doctor and a CCP, by helicopter. Two additional pre-hospital critical care teams were requested from areas with shared geographic borders as mutual aid: One helicopter team from LAA, with two doctors and a CCP, and a fast response car from HIOWAA with one doctor and two CCPs. In addition, two BASICS doctors also attended the scene, one of whom acted as the Medical Incident Advisor.

The scene was an urban park where three patients were confirmed to be in TCA as a result of stab injuries. A further three patients had also suffered stab injuries. By the time the incident had concluded three patients were pronounced dead at the scene. Two patients were transported to the local trauma unit and one patient was transported to the regional Major Trauma Centre depending on the severity of their injuries.

The incident was conducted against the backdrop of a global pandemic of the novel coronavirus COVID-19, requiring that all casualties received care from clinicians who were wearing enhanced levels of PPE. For casualties requiring potential aerosol generating procedures (AGPs), which include airway management and surgical interventions, this PPE consists of a FFP3 mask or respirator, goggles or visor, a fluid repellent one-piece white suit with a hood and multiple layers of gloves (Level 3 PPE). The suits bear no form of identification of the wearer, so some clinicians had attached A5 sized stickers to identify clinicians by name and role, others bore no identification. At the height of the incident there were approximately 20 clinicians from 5 services all wearing level 3 PPE, most of whom were in identical UK standard issue “Tyvek” brand white suits.

## Method

In order to identify good practice and areas for improvement, we conducted a semi-structured telephone interview with critical care clinicians who attended the scene and the coordinating TVAA CCP from the SCAS EOC HEMS desk. The interviews were focused specifically on the challenges of providing care to multiple stab victims whilst wearing COVID-19 PPE, rather than the wider clinical, logistical and operational challenges of responding to such an incident.

The following questions were discussed:

- *Describe your job role on the 20th June*
- *Describe the number of major incidents you have previously attended*
- *When you were dispatched what sort of scene were you expecting?*
- *What preparations did you make on route? Specifically briefing/donning suits/allocating roles*
- *What extra information or direction were you given on arrival at scene? eg from scene commander.*
- *Briefly describe the scene on arrival.*
- *On arrival at scene what PPE was being worn?*
- *What PPE was worn by other clinicians dealing with the same patient?*
- *How did this change as the incident progressed?*
- *What clinical interventions did you conduct?*
- *What was the disposition of your patient? (Prononced life extinct at the scene or conveyed to hospital)*
- *What particular challenges did you find regarding communication: within the team/to external teams/to scene command?*
- *What particular challenges did you find regarding situational awareness?*
- *What went well and where was there scope for improvement.*
- *From the aspect of conducting a major incident in level 3 PPE what might you advise others attending a similar event?*
- *From the aspect of conducting a major incident in level 3 PPE what might you do differently if attending a similar event in future?*

## Results

Nine clinicians were interviewed, from TVAA, LAA, HIOWAA and BASICS, who had varying levels of experience of responding to mass casualty events. Table 1 shows the challenges that were reported and the solutions proposed by the interviewees.

**Table 1:** Challenges, good practice and proposed solutions

Challenge or good practice identified	Proposed solutions
Good practice – South Central Ambulance Service (SCAS) senior commander was wearing an easily recognisable tabard and didn’t require level 3 PPE	Only clinicians conducting Aerosol Generating Procedures (AGPs) should be in level 3 Personal Protective Equipment (PPE). Develop better labelling or supply scene tabards for all clinicians. (see below)
It was difficult to identify clinicians by role and the labels used for identification were insufficient.	In order to distinguish skill levels and grades for clinicians wearing Level 3 PPE it is recommended that a colour coded tabard similar to that used by scene commanders be developed. This would identify Critical Care doctors and Critical Care Paramedics (CCPs) and be standardised across the UK as per the National Ambulance Resilience Unit guidance (Appendix 1) This tabard could either be disposable or washable at 60 °C to facilitate infection control.
Once a fluid repellent suit is donned it makes it impossible to access items in the pockets of clothes underneath. This included radios and car keys.	The senior commander carries a drop box to collect the keys of response vehicles from Critical Care teams as they arrive, allowing these vehicles to be moved as necessary.
Carrying and using radios whilst wearing level 3 PPE is challenging	Use earpieces with a push to talk facility or a belt to carry the radio externally.
The wearing of masks and hoods meant it was difficult to hear verbal instructions	Closed loop communication would be beneficial
It was difficult to send and receive updates from the HEMS desk once level 3 PPE was donned. It was commented that fewer updates from scene were received that would be usual from an non-COVID incident	The teams that arrived at the scene later, when most interventions had already been undertaken, should have one clinician don full level 3 PPE, with at least one other remaining in level 2 PPE to facilitate communication and situational awareness. A windscreen report is a brief summary of what can be seen immediately on arrival at a large or major incident; they are particularly valuable when taken from a helicopter above the scene and can be done by taking an aerial scene photograph to refer to later. The importance of a windscreen report is increased when later communications may be impaired by the use of PPE.
There are multiple challenges regarding communication and situational awareness	Training. Organisations should train for multi-casualty situations wearing PPE. To-date, the majority of training has focused on individual patient care whilst wearing PPE. CRM. Individuals should be aware of how PPE compromises recognition and communication. As a result, it takes time to establish what has occurred, who is on the scene and what their role is.
Some teams carried large storage bags containing a variety of sizes of PPE and it was time-consuming to find the correct sizes.	Clinicians could carry one set of PPE of the correct size for the entire shift, with resupply from a stock holdall as required.

## Discussion

As a result of the interview the common themes that developed were:

- When dispatched a number of crews were expecting a single victim. Updates on route proved key to developing an understanding of the scene.
- In teams that arrived by vehicle the co-driver was able to don PPE whilst on route to the scene and prepare the driver’s PPE. In teams that arrived by aircraft one member of the team was identified to don level 3 PPE first and was assisted by another crew member.
- The casualties in TCA were fortunately all situated in close proximity. This facilitated the first critical care team on scene to receive a handover from the SCAS teams already in attendance and then to conduct further clinical assessments and multiple interventions as required in rapid succession.
- The initial response of the first critical care team on scene was to support the SCAS ambulance crews already on scene who were only in level 2 PPE and therefore had not been able to undertake airway interventions.
- It was difficult to identify individual clinicians in COVID-19 level 3 PPE by role as the stickers used for identification were insufficient (a number of respondents reported being unable to recall any name badges being worn, implying that those worn were insufficiently prominent). This challenge was exacerbated with clinicians from different organisations where individuals could not be recognised from their voice and mannerisms. Several respondents reported individual identification together with recognising the clinician’s role whilst in Level 3 PPE as the greatest challenge of the incident.
- The inability to identify individuals by name or role led to repeated assessments and reviews of the patients as each critical care team arrived. Even when an exchange occurred between two clinicians it was then difficult to identify an individual again to continue the interaction.
- The wearing of hoods and FFP3 face masks severely compromised the usual non-verbal communication techniques as well as necessitating a raised voice, both of which led to misinterpretation of the tone and intent of a conversation.

## Conclusions

We conducted a semi structured telephone interview with the critical care clinicians who were involved in the incident. The interviews focused specifically on the challenges of responding whilst wearing COVID-19 PPE, rather than the wider challenges of responding to such an incident. The key learning points identified were:

- Improving the identifiability of clinicians in level 3 PPE
  - wearing identification tabards
  - using visible labelling on PPE suits
- Improving communication by radio
  - using a belt to carry the radio
  - using an earpiece and push to talk system.
- Training in conducting multi casualty incidents in level 3 PPE.

## Data Availability

The datasets generated and analysed during the current study are not publicly available due clinician confidentiality but an anonymised redaction would be available from the corresponding author on reasonable request.

## Appendix

Command Tabard designs are outlined on page 51 of the NARU guidance.

https://naru.org.uk/wp-content/uploads/2016/09/NARU-CC-GUIDANCE-V.1.2.OCT-2015jm.pdf

A suitable design for critical care teams would be based on the colour scheme of the Doctor tabard and read “Critical Care Doctor” and “Critical Care Paramedic”.

## Abbreviations

AGP: Aerosol Generating Procedure
BASICS: British Association for Immediate Care
CCP: Critical Care Paramedic
CRM: Crew Resource Management (Human Factors)
EOC: Emergency Operations Centre
FFP3: Filtering Face Mask
HEMS: Helicopter Emergency Medical Service
HIOWAA: Hampshire and Isle of Wight Air Ambulance
LAA: London’s Air Ambulance
PPE (Level 2/3): Personal Protective Equipment
SCAS: South Central Ambulance Service
TCA: Traumatic Cardiac Arrest
TVAA: Thames Valley Air Ambulance
